# Inhibitory Efficacy of Medicinal Plant Extracts from the Family Acanthaceae Against Skin Pathogenic Bacteria, Antioxidant and Anti-Inflammatory Activities

**DOI:** 10.3390/plants15172650

**Published:** 2026-08-29

**Authors:** Kullanun Mekawan, Sureeporn Suriyaprom, Nitsanat Cheepchirasuk, Saranya Chaiwaree, Hans Bäumler, Yingmanee Tragoolpua

**Affiliations:** 1 Department of Biology, Faculty of Science, Chiang Mai University, Chiang Mai 50200, Thailand; kullanun_me@cmu.ac.th (K.M.); sureeporn.suriyaprom@cmu.ac.th (S.S.); nitsanat_cheep@cmu.ac.th (N.C.); 2 Office of Research Administration, Chiang Mai University, Chiang Mai 50200, Thailand; 3 Department of Pharmaceutical Technology and Biotechnology, Faculty of Pharmacy, Payap University, Chiang Mai 50210, Thailand; saranya_c@payap.ac.th; 4 Institute of Transfusion Medicine, Charité-Universitätsmedizin Berlin, 10117 Berlin, Germany; 5 Natural Extracts and Innovative Products for Alternative Healthcare Research Group, Faculty of Science, Chiang Mai University, Chiang Mai 50200, Thailand

**Keywords:** anti-bacteria, anti-inflammatory activity, antioxidant, family Acanthaceae

## Abstract

Medicinal plants in the Acanthaceae family have long been used in Thai traditional medicine. In this study, ethanolic extracts of four plant species, *Andrographis paniculata*, *Thunbergia laurifolia*, *Acanthus ebracteatus*, and *Rhinacanthus nasutus*, were prepared and their phytochemical and pharmacological properties were evaluated. Total phenolic and flavonoid content assays were used to investigate phytochemical content and phytochemical profiles were characterized with LC-MS, while antioxidant activities were assessed via colorimetric methods. The extracts were also tested for antibacterial activity against several skin pathogens, such as *Pseudomonas aeruginosa*, *Cutibacterium acnes*, *Micrococcus luteus*, *Staphylococcus aureus*, and methicillin-resistant *S. aureus*, using agar well diffusion and broth dilution methods. Cytotoxic effects on the RAW 264.7 macrophage cell were assessed using the MTT assay. The extracts were further evaluated for anti-inflammatory activity in an LPS-stimulated RAW 264.7 cell. Inflammatory gene expression was assessed by quantifying the mRNA levels of *iNOS*, *COX-2*, *TNF-α*, *IL-1β*, *IL-6*, and *NF-κB* genes using RT-qPCR. The results revealed that among the four species, *T. laurifolia* extract showed the highest phenolic and flavonoid levels, consistent with the greatest antioxidant activity. Moreover, LC-MS profiling annotated a range of secondary metabolites in the extracts. The inhibition zones were observed for all plant extracts when tested against the skin pathogenic bacteria except *C. acnes*, and *A. paniculata* did not inhibit *P. aeruginosa*, indicating the antibacterial activity of the extracts. The minimum inhibitory concentration (MIC) and minimum bactericidal concentration (MBC) values differed across the extracts and bacterial strains. *T. laurifolia* exhibited the most potent activity against various bacterial pathogens, while *A. paniculata* was most effective against *C. acnes* at 0.98 mg/mL. In addition, all plant extracts reduced nitric oxide production and downregulated the expression of inflammatory genes. Among all plant species, *A. paniculata* showed the most potent activity with the highest NO inhibition of 65.68% at 60 µg/mL. Therefore, these findings support the antioxidant, antibacterial, and anti-inflammatory potential of *A. paniculata*, *T. laurifolia*, *A. ebracteatus* and *R. nasutus*, which are traditionally used in folk medicine.

## 1. Introduction

Skin is an important organ that protects the body from the outside environment such as physical agents, bacteria, fungi, viruses, and protozoa. Among these, bacteria are major pathogens and responsible for various skin infections with clinical manifestations depending on factors such as the site of infection and the host immune status. Common skin-associated pathogenic bacteria include Gram-positive species such as *Staphylococcus aureus*, *Micrococcus luteus*, *Cutibacterium acnes*, and methicillin-resistant *S. aureus* (MRSA). Moreover, Gram-negative bacteria, e.g., *Proteus mirabilis*, *Escherichia coli* and *Pseudomonas aeruginosa,* may cause skin infection from participation in various activities that involve soil or water. These microorganisms can enter the body through mucosa, cuts, open wounds, or other disruptions of the skin barrier. The examples of the skin infections include impetigo, staphylococcal scalded skin syndrome, erysipelas, cellulitis, folliculitis, furuncles, carbuncles, and fasciitis. Notably, normal flora can also play a protective role by inhibiting the colonization of pathogenic microorganisms [1].

Upon bacterial invasion, the host immune system initiates an inflammatory response characterized by pain, heat, redness, swelling, and loss of function. Inflammation is a crucial defense mechanism that protects the host against infection and injury [2]. However, excessive or chronic inflammation cannot only lead to tissue damage and increase the severity of wounds but also to impaired B-cells due to accumulation of atypical memory B-cells [3]. In addition, bacterial infection and inflammation can stimulate the production of oxidants that promote cascade reactions and cause oxidative damage to the cells [4]. Although synthetic drugs and antibiotics have been used to cure skin infections by inhibiting the growth of bacteria, prolonged usage may contribute to the development of antimicrobial resistance. For example, methicillin-resistant *Staphylococcus aureus* (MRSA) is resistant to methicillin. In addition, the side effects and high cost of synthetic drugs highlight the need for alternative therapeutic agents.

Natural products derived from plants have been widely used for treating human diseases for a long time. Medicinal plants produce a diverse array of secondary metabolites including phenolic acids, flavonoids, tannin, lignin, and other small compounds. Numerous studies report that plant phytochemicals have pharmacological activities. Therefore, plant extracts represent a promising, alternative to synthetic drugs due to their effectiveness, availability, and cost-efficiency [5].

The family Acanthaceae is one of the families in dicotyledonous flowering plants that widely distributed in tropical and subtropical regions such as China, Japan, India, and Thailand [6]. In Thailand, Acanthaceae were commonly used as traditional medicine for treating fever, piles, and skin disease. Several species, including *Andrographis paniculata*, *Clinacanthus nutans*, *Graptophyllum pictum*, and *Ruellia brittoniana*, have been used as medicinal plants with great therapeutic potency and played an important role in anti-cancer, antioxidant, anti-inflammatory, anti-hypoxic, neuroprotective, anti-proliferative, anti-diarrheal, anti-fungal, and antiparasitic activities [7,8]. These pharmacological properties are largely attributed to their composition, which is rich in phytochemicals including phenolics, flavonoids, alkaloids, lignans, saponins, triterpenoids, steroids, and fatty acids [9]. In Thailand, more than 40 genera and over 230 species of Acanthaceae have been identified. Among them, *Andrographis paniculata* (AP), *Thunbergia laurifolia* (TL), *Acanthus ebracteatus* (AE), and *Rhinacanthus nasutus* (RN) are widely used in Thai traditional medicine. These plants are commonly utilized in their whole form for disease prevention and treatment due to their rich content of bioactive compounds. Previous studies reported that major phytochemicals in AP, AE, and RN are andrographolide, verbascoside, and naphthoquinones, respectively, while TL contains apigenin, caffeic acid, and gallic acid. These bioactive compounds display therapeutic properties such as antimalarial, antidiabetic, antibacterial, and anti-fungal activity [10,11,12]. These four species were selected because they share a common use in Thai traditional medicine for treating skin diseases. However, previous studies on AP, TL, AE, and RN have been conducted independently, which precludes direct comparison of their relative potency. Consequently, the combined antibacterial, antioxidant, and anti-inflammatory activities of these four Acanthaceae species have not been systematically compared. Thus, comparing biological activity under the same conditions enables the prioritization of plant species for selection of future product development. RAW 264.7 macrophages were selected as the cellular model in this study because this cell line is one of the most commonly used as models for screening anti-inflammatory and immunomodulatory compounds. In addition, RAW 264.7 cells are commercially available, easy to culture, and can be readily expanded for screening tests. When comparing RAW 264.7 cells to primary cells such as peripheral blood mononuclear cells (PBMCs), the study using RAW 264.7 cell line may reduce donor-to-donor variability. Many studies have shown these cells exhibit a robust inflammatory response to LPS stimulation, including NO production and pro-inflammatory mediator expression [13,14].

Therefore, this study aims to examine the efficacy of four plant extracts from the family Acanthaceae for their total phenolic and flavonoid contents, antioxidant capacity, antibacterial activity against skin pathogenic bacteria as well as anti-inflammatory activity in the RAW 264.7 macrophage cell line.

## 2. Results

### 2.1. Yields of Medicinal Plant Extracts, Total Phenolic and Flavonoid Contents of Four Selected Species of the Family Acanthaceae

Medicinal plants in family Acanthaceae including *Andrographis paniculata* (AP), *Thunbergia laurifolia* (TL), *Acanthus ebracteatus* (AE), and *Rhinacanthus nasutus* (RN) were extracted by 95% ethanol. The extraction yields of ethanolic extracts from four plant species are shown in Table 1 with the highest yield of 6.68% from AP.

Total phenolic contents (TPC) and total flavonoid contents (TFC) were evaluated by using Folin–Ciocalteu reagent and aluminum chloride hexahydrate reagent, respectively. Total phenolic content of the extracts expressed as milligrams of gallic acid equivalents (GAE) ranged from 18.33 ± 1.08 to 153.97 ± 1.55 mg GAE/100 g extract, while the content of flavonoid expressed as quercetin equivalents ranged from 12.89 ± 1.04 to 60.17 ± 0.87 mg QE/100 g extract (Table 2). TL showed the highest TPC, followed by AE, RN, and AP. A similar trend was observed for TFC, with TL showing the highest value, followed by RN, AE, and AP.

### 2.2. Liquid Chromatography–Mass Spectrometry (LC-MS) Analysis

**Figure 1:**
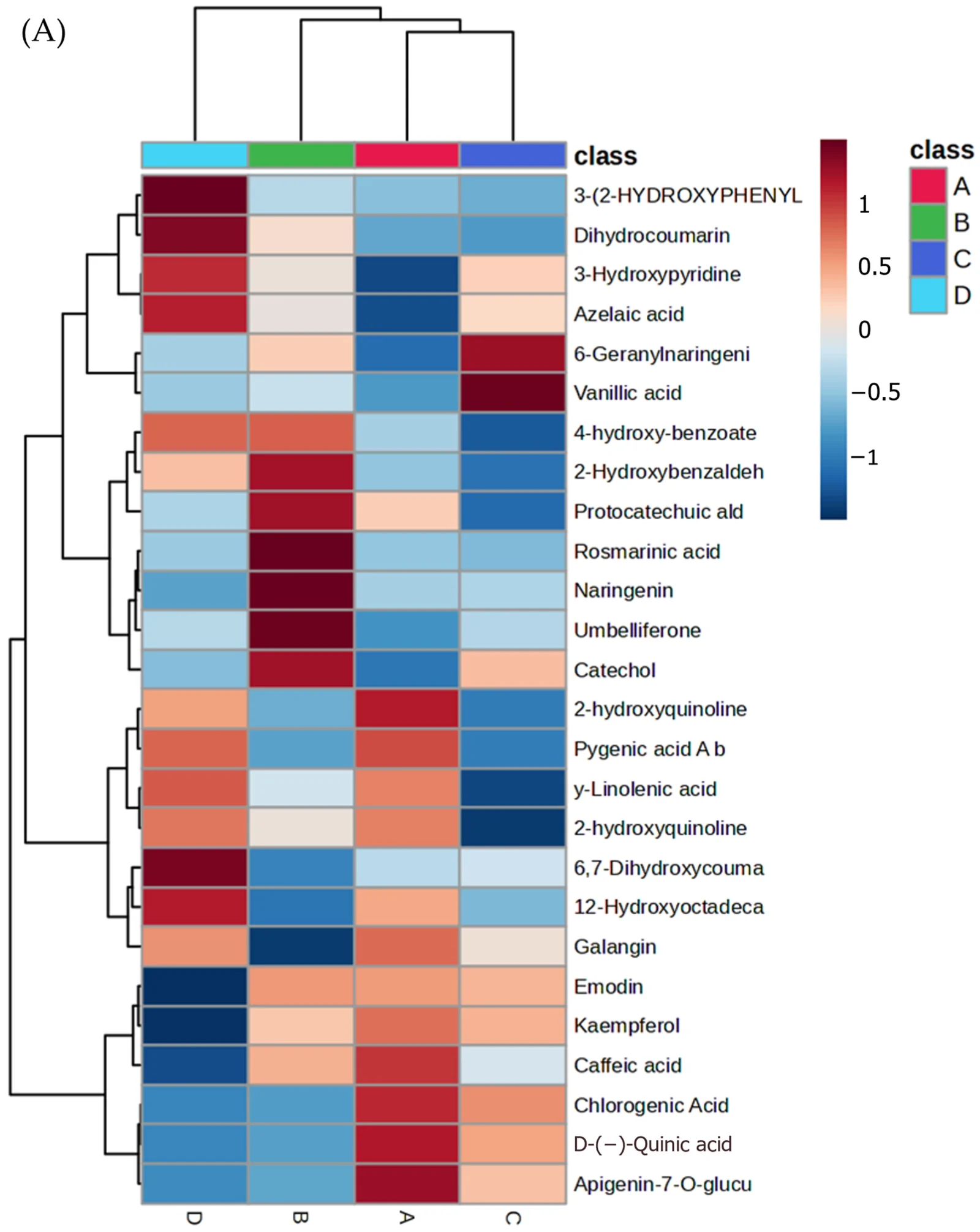

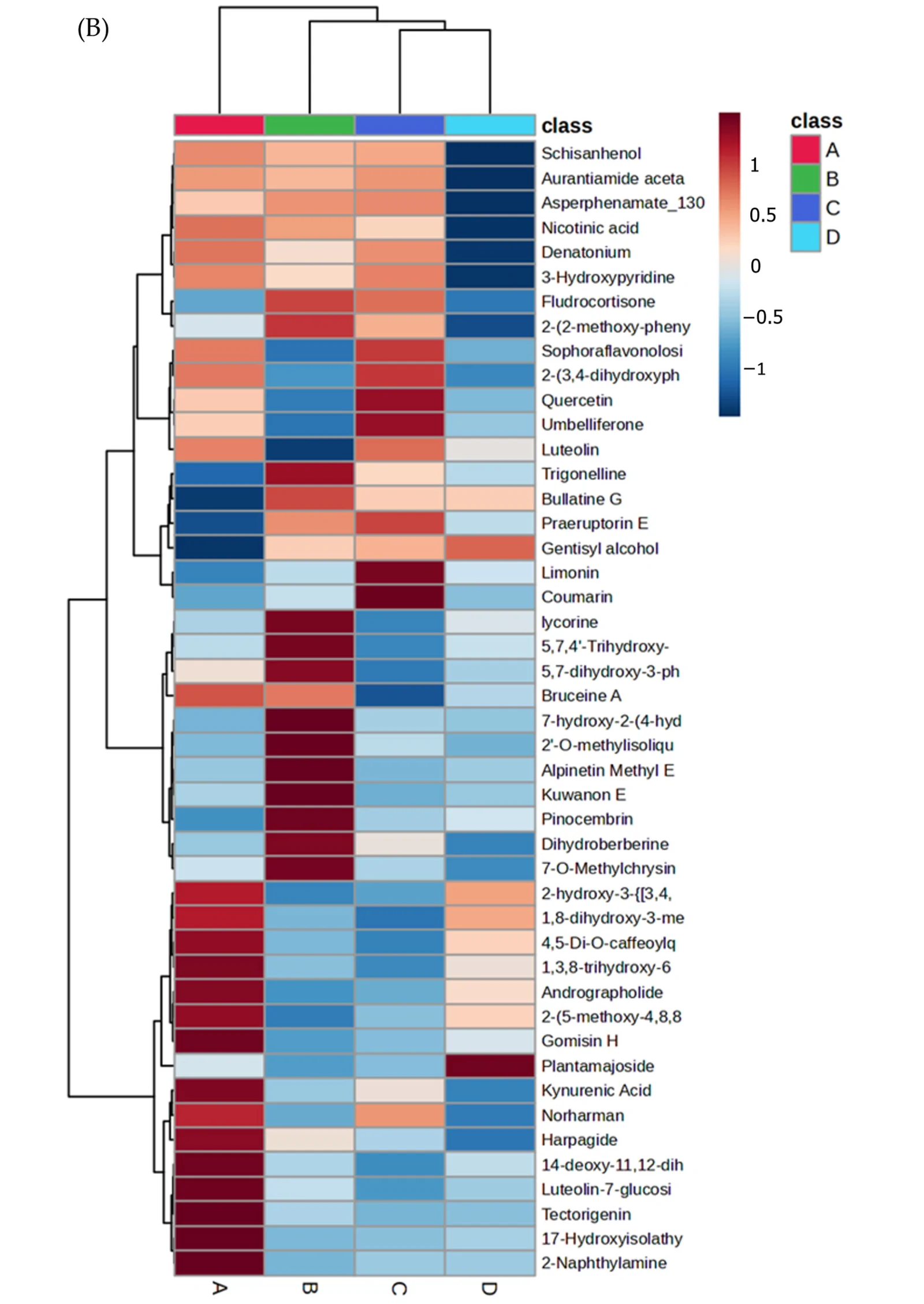
Heat map of LC-MS annotated metabolites in Acanthaceae plant extracts in negative (**A**) and positive (**B**) ionization modes. Rows correspond to individual annotated metabolites, and columns correspond to the four Acanthaceae species including AP (A), TL (B), AE (C), and RN (D). The horizontal and vertical lines represent the dendrograms generated from hierarchical clustering analysis (HCA), indicating the chemical profile similarities among plant samples (top) and expression patterns among metabolites (left), respectively. The top annotation bar (“class”) identifies each sample column by color, which is defined in the right-corner legend panel. The color scale indicates the relative abundance of metabolites based on Z-score normalization, ranging from deep red (relatively high abundance) to deep blue (relatively low abundance).

The chemical profiles of four Acanthaceae extracts were characterized using liquid chromatography–mass spectrometry (LC-MS) analysis. Chromatographic analysis in both positive and negative ionization modes revealed a diverse array of metabolites with distinct retention times across the four plant extracts. Figure 1 shows a total of 97 putative metabolites (66 in positive mode and 31 in negative mode) were tentatively annotated across both ionization modes, all with high-confidence annotation scores (score ≥ 1.70), indicating reasonable confidence in the putative annotations (Tables S1 and S2). In the negative ionization mode analysis (Figure 1A), TL showed notably high relative abundance for several phenolic compounds in the upper cluster such as rosmarinic acid, naringenin, umbelliferon, and protocatechuic aldehyde. In contrast, AP exhibited the highest relative abundance of compounds in the lower cluster, particularly chlorogenic acid, caffeic acid, D-(−)-quinic acid, and apigenin-7-O-glucoside. AE and RN displayed moderate relative abundance across most detected metabolites with dominant compounds such as 6-Geranylnaringenin and vanillic acid in AE, and 6,7 dihydroxycoumarin in RN.

In the positive ionization mode (Figure 1B), AP exhibited the highest relative abundance of compounds in the lower cluster, especially andrographolide, 14-deoxy-11,12-dihydroandrographolide, harpagide, norharman, kynurenic acid, luteolin-7-glucoside, tectorigenin, and 17-Hydroxyisolathyrol. TL demonstrated high relative abundance for several polyphenols in the middle cluster, for instance coumarin, lycorine, 5,7,4′-Trihydroxyflavone, bruceine A as well as dihydroberberine. In contrast, AE showed high relative abundance in the upper cluster, particularly schisanhenol, aurantiamide acetate, and asperphenamate. RN displayed the lowest relative abundance across most detected metabolites, with plantamajoside annotated as a major compound. Overall, AE and RN showed comparatively lower relative abundance across the detected metabolite profiles compared to AP and TL.

### 2.3. Antioxidant Activities of Four Selected Species of the Family Acanthaceae

The antioxidant activity of four plant species was evaluated using 2,2-Diphenyl-1-picrylhydrazyl (DPPH) radical scavenging activity and 2,2′-azino-bis (3-ethylbenzothiazoline-6-sulfonic acid) (ABTS) radical cation decolorization assay. For both assays, results were reported as IC_50_ values, representing the concentration of each extract required to neutralize 50% of the initial free radicals. As shown in Table 3, TL extract exhibited the strongest DPPH radical scavenging activity, with an IC_50_ of 0.16 ± 0.02 mg/mL, and the most potent ABTS radical cation decolorization activity, with an IC_50_ of 0.76 ± 0.07 mg/mL. Consistently, TL also demonstrated the highest antioxidant capacity in both assays, with values of 85.09 ± 11.70 mg TAE/g extract for DPPH and 15.71 ± 1.34 mg TAE/g extract for ABTS.

### 2.4. Antibacterial Activity of Four Selected Species of the Family Acanthaceae

#### 2.4.1. Determination of Antibacterial Activity from Four Selected Species of the Family Acanthaceae

The agar well diffusion method was used to evaluate the antibacterial activity of plant extracts against skin pathogenic bacteria, including *P. aeruginosa*, *C. acnes*, *M. luteus*, *S. aureus*, and MRSA. The diameter of the inhibitory zones was measured to assess antibacterial efficacy (Table 4). The results revealed that TL, AE, and RN inhibited the growth of the tested bacteria, with inhibition zone diameters ranging from 10.8 to 22.3 mm, except against *C. acnes*. In contrast, AP did not produce a clear zone of inhibition against *C. acnes* or *P. aeruginosa*, but exhibited the greatest inhibitory effect against *M. luteus* (23.5 ± 0.6 mm).

#### 2.4.2. Determination of Minimum Inhibitory Concentration (MIC) and Minimum Bactericidal Concentration (MBC) of Extracts of Four Selected Species of the Family Acanthaceae for Antibacterial Activity

The broth microdilution method was used to determine the minimum inhibitory concentration (MIC) and minimum bactericidal concentration (MBC) of the extracts. The extracts showed MIC/MBC at concentrations between 0.98 and 125.00 mg/mL after testing with skin pathogenic bacterial species (Table 5). AE showed the highest activity against *P. aeruginosa* with MIC and MBC values of 15.63 mg/mL. TL and AP extract exhibited the highest activity against *S. aureus* and *C. acnes*, with MIC and MBC values of 3.91 and 0.98 mg/mL, respectively. In the case of *M. luteus* and MRSA, more than one extract demonstrated inhibitory effects at the same lowest concentration. TL, AE, and RN inhibited the growth of *M. luteus* at a concentration of 31.25 mg/mL. Similarly, MRSA was inhibited by TL and AE at the lowest concentration of 7.81 mg/mL. In contrast, AP extract demonstrated the lowest activity against all tested bacteria, except in *C. acnes*. Gentamicin, used as a positive control, was tested against *P. aeruginosa*, *C. acnes*, *M. luteus*, and *S. aureus*, with MIC and MBC values ranging from 0.49 to 1.95 µg/mL. Vancomycin was used as a positive control for MRSA, with MIC and MBC values of 0.98 µg/mL.

### 2.5. Anti-Inflammatory Activity of Four Selected Species of the Family Acanthaceae

#### 2.5.1. Effect of Extracts of Four Selected Species of the Family Acanthaceae on Cell Viability of RAW 264.7 Cells

**Figure 2:**
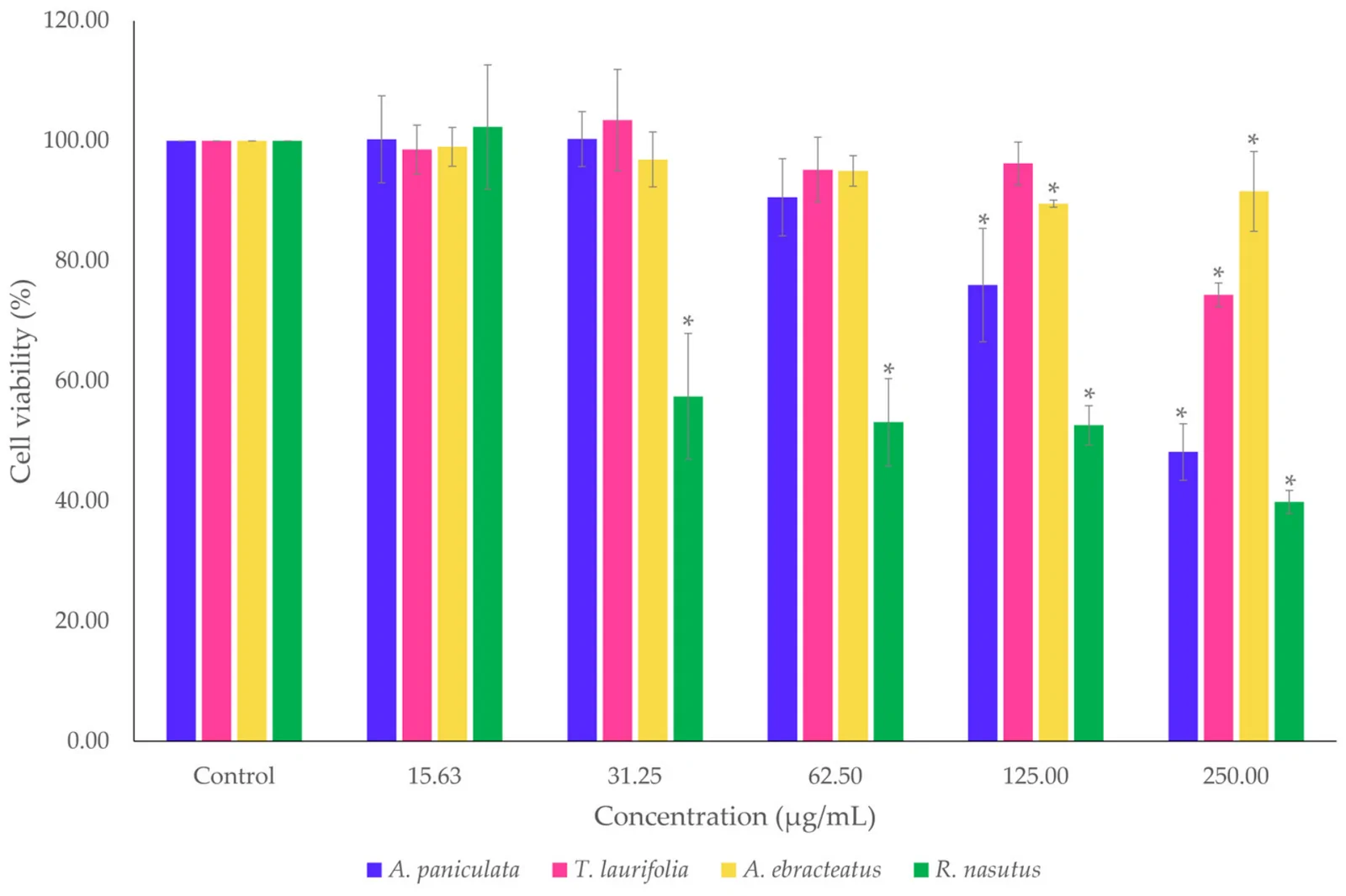
Effects of Acanthaceae plant extracts on RAW 264.7 cell viability. The results were expressed as means  ±  standard deviation (SD) from three independent experiments, compared with the control group. * *p*  <  0.05 vs. control group.

The 3-(4,5-Dimethylthiazol-2-yl)-2,5-Diphenyltetrazolium Bromide (MTT) assay was used to evaluate the cytotoxicity of the extract in RAW 264.7. Cells were treated with Acanthaceae extracts at concentrations of 15.63, 31.25, 62.50, 125, and 250 µg/mL for 24 h (Figure 2). The results showed that AP and AE extracts significantly reduced cell viability at concentrations of 125 and 250 µg/mL, while TL exhibited cytotoxic effects at 250 µg/mL. In addition, RN exhibited cytotoxic effects at 31.25 µg/mL. Based on these findings, non-cytotoxic concentrations were selected for subsequent experiments. Specifically, AP, TL, and AE were used at 60, 30, and 15 µg/mL, whereas RN was used at 15, 7.5, and 3.75 µg/mL.

#### 2.5.2. Inhibition of Nitric Oxide Production in LPS-Stimulated RAW 264.7 Cells

**Figure 3:**
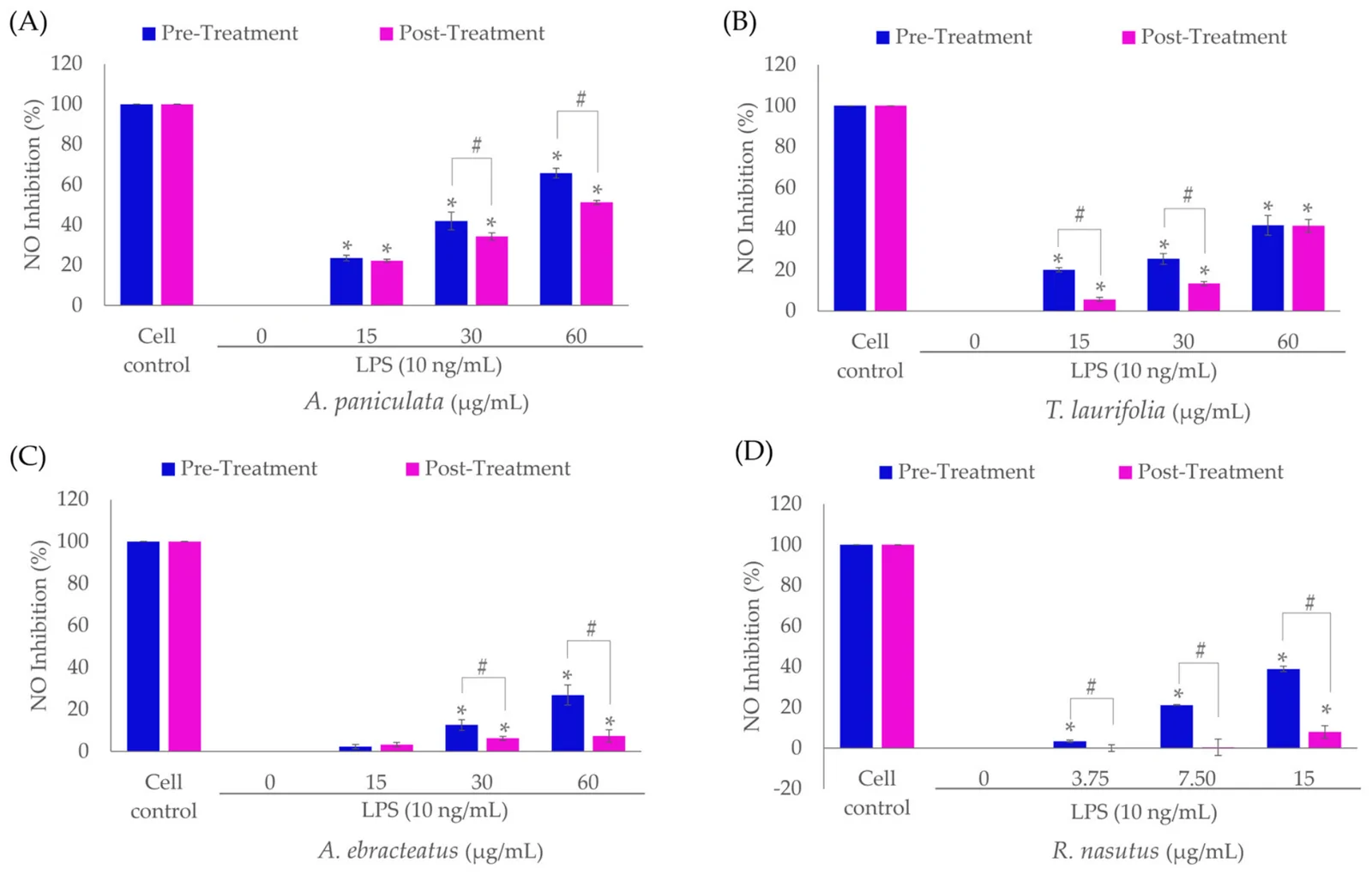
Effects of the Acanthaceae extracts on nitric oxide inhibition in LPS-stimulated RAW 264.7 cells: (**A**) AP; (**B**) TL; (**C**) AE; (**D**) RN. The results were expressed as means  ±  standard deviation (SD) (*n* = 3), as determined by the Griess assay. * Significant difference (*p* < 0.05) compared with the LPS group; # significant difference (*p* < 0.05) between pre- and post-LPS stimulation.

The effects of four Acanthaceae plant extracts on nitric oxide (NO) inhibition were evaluated in LPS-stimulated RAW 264.7 macrophage cells using pre-treatment and post-treatment approaches, as determined by the Griess assay (Figure 3). The results indicate that all plant extracts significantly reduced NO production in a dose-dependent manner under both pre- and post-treatment conditions. Notably, the percentage of NO inhibition was significantly higher in the pre-treatment group compared to the post treatment (*p* < 0.05). Among the tested extracts, AP showed strongest NO inhibitory activity, with inhibition rates of 65.68% and 51.25% in pre-treatment and post-treatment, respectively. This was followed by TL (41.70%), RN (38.97%), and AE (26.90%) at the highest tested concentrations under pre-treatment conditions.

#### 2.5.3. Effects of Extracts of Four Selected Species of the Family Acanthaceae on the mRNA Expression of Inflammatory Mediators in LPS-Stimulated RAW 264.7

The effects of extracts of the family Acanthaceae on the mRNA expression of inflammatory genes in LPS-stimulated RAW 264.7 cells were evaluated using real-time quantitative PCR. The results showed that Acanthaceae extracts significantly downregulated the mRNA expression of six inflammatory genes including *iNOS*, *COX-2*, *IL-1β*, *IL-6*, *TNF-α*, and *NF-κB* under both pre-treatment and post-treatment models.

**Figure 4:**
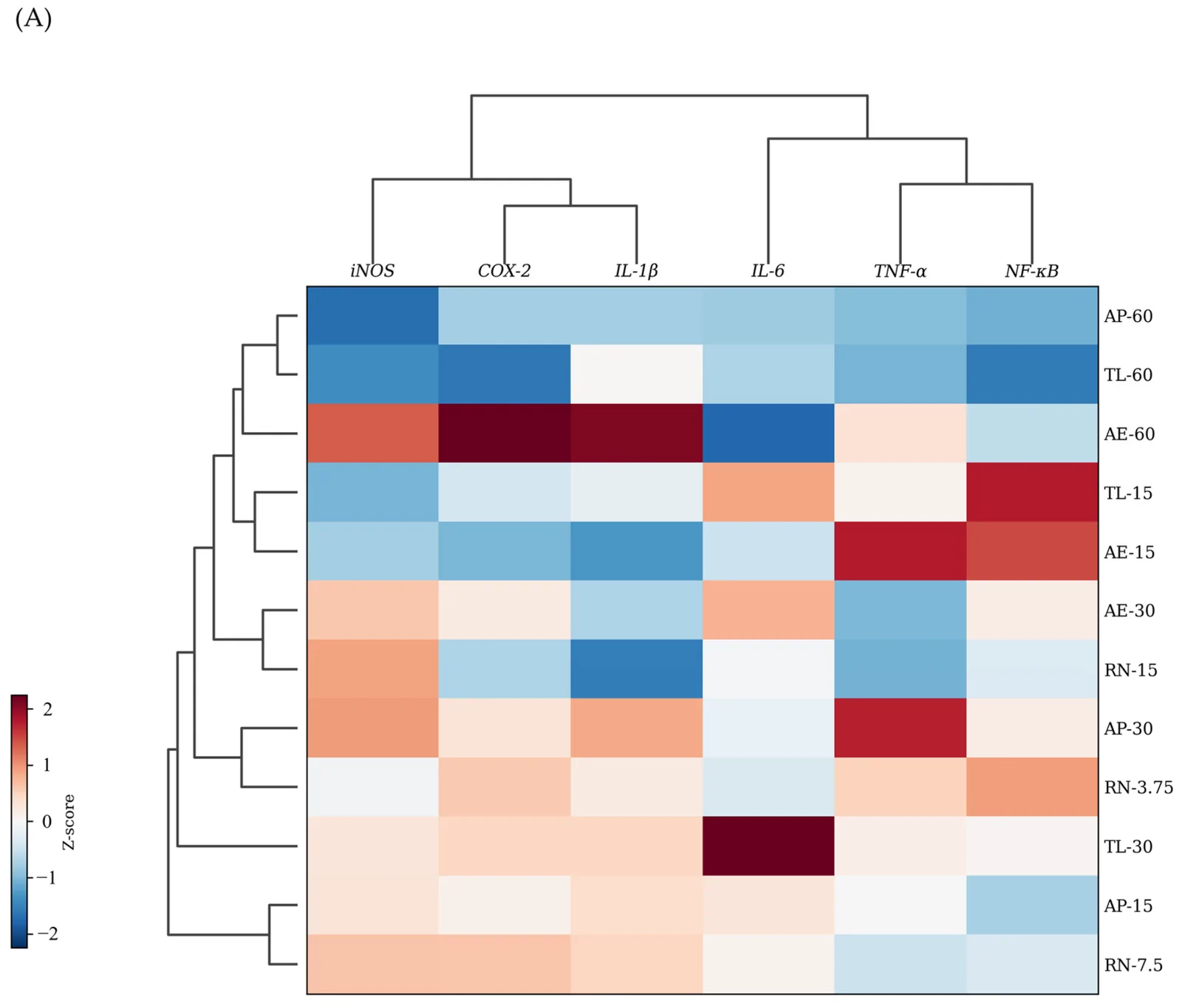

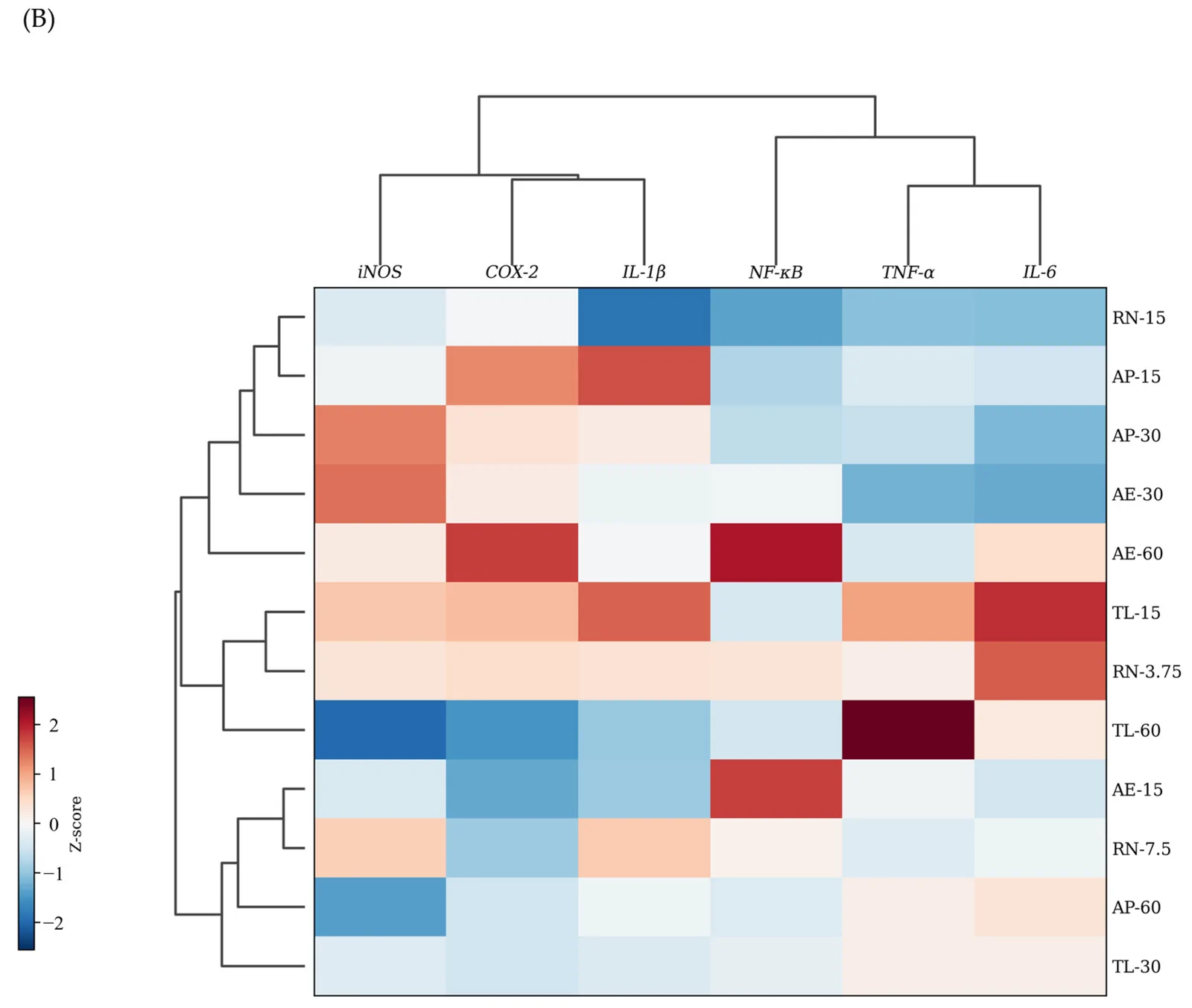
Hierarchical clustering heatmap of inflammatory gene expression in LPS-stimulated RAW 264.7 cells. Clustering was performed using Ward’s linkage method with Euclidean distance based on Z-score normalized data for (**A**) pre-treatment and (**B**) post-treatment conditions. Dendrogram illustrates similarities in gene expression profiles among treatment groups and target genes.

Hierarchical clustering analysis of gene expression profiles was performed using Ward’s linkage method with Euclidean distance based on Z-score normalized data (Figure 4). The heatmap visualization shows that red indicates relatively higher expression and blue indicates relatively lower expression compared to the mean across all treatments. The dendrogram illustrates similarities in gene expression profiles among treatment groups and target genes.

Gene clustering analysis in both conditions revealed that *COX-2*, *IL-1β*, and *iNOS* were grouped into the same cluster, indicating similar suppression patterns across treatment. In contrast, *IL-6*, *TNF-α*, and *NF-κB* formed a separate cluster, suggesting differential regulation by the extracts. In pre-treatment, higher extract concentration generally resulted in greater suppression of gene expression, with AP extract at a concentration of 60 µg/mL (AP-60) and TL at a concentration of 60 µg/mL (TL-60) exhibiting the strongest effects. In contrast, in the post-treatment model, RN at a concentration of 15 µg/mL (RN-15) showed the best inhibition of gene expression across all treatments. Notably, increased extract concentration did not consistently correlate with enhanced suppression of inflammatory gene expression.

## 3. Discussion

In Thailand, plants belonging to the family Acanthaceae are widely used in traditional medicine [15]. Plant extracts are rich in phytochemicals, including phenolics, terpenoids, alkaloids, anthocyanins, tannins and lignans, which exhibit a wide range of biological properties such as antioxidant, anti-inflammatory, antimicrobial, antihypertensive, and anti-cancer [16]. In this research, variations in bioactive compound content were observed among the four Acanthaceae extracts. TL exhibited the highest TPC (153.97 ± 1.55 mg GAE/100 g extract) and TFC (60.17 ± 0.87 mg QE/100 g extract), consistent with previous reports describing the abundance of flavonoids and hydroxycinnamic acid derivatives in this species [17]. Correspondingly, TL demonstrated the strongest radical scavenging activity in both DPPH (IC_50_ = 0.16 ± 0.02 mg/mL) and ABTS (IC_50_ = 0.76 ± 0.07 mg/mL) assays. AE and RN showed intermediate phenolic contents and antioxidant activities, while AP recorded the lowest TPC and TFC, along with the weakest antioxidant activity (DPPH IC_50_ = 0.67 ± 0.08 mg/mL). The observed antioxidant capacity of the Acanthaceae extracts correlated with their phenolic composition, as these compounds can directly scavenge free radicals and chelate metal ions.

Furthermore, LC-MS analysis revealed that the metabolite profiles of the four Acanthaceae species were generally consistent with previous reports. In AP, earlier studies identified compounds such as octadecanoic acid, stigmasterol, phenanthrene carboxylic acid, 7-ethenyl 9-octadecenoic acid, geranyl-α-terpinene, 13,15-octacosadiyne, methyl 8,10-octadecadiynoate, and stigmast-5-en-3-ol, octadecanoate (3β) [18]. In addition, andrographolide has been reported as the major bioactive compound in AP, along with related diterpenoids such as 14-deoxy-11,12-didehydroandrographolide, andrograpanin, and neoandrographolide [19,20,21].

For TL, previous studies have identified rosmarinic acid as a major phytochemical, a phenolic ester derived from caffeic acid and 3,4-dihydroxyphenylacetic acid [17]. This is consistent with the present study, in which rosmarinic acid was also detected in TL extracts. In the case of AE extract, verbascoside has been reported as a major active compound in ethanolic extracts [12], whereas it is often absent in non-polar extracts such as hexane. Interestingly, although the present study employed a similar extraction solvent, verbascoside was not detected by LC-MS analysis [22]. The metabolite profile of RN extract observed in this study also aligns with previous reports such as vanillic acid. In addition, RN has been reported to contain napthoquinones such as Rhinacanthin A, B, C, D, G, H, I, J, K, L, M, N, O, P, and Q, as well as α-lapachone, which have been isolated from its leaves and roots [23,24]. According to the previous literature, the metabolites in each plant species may account for the antibacterial, antioxidant, and anti-inflammatory activities that were observed in this study. Andrographolide in *Andrographis paniculata* has been reported to disrupt bacterial cell wall and nucleic acid biosynthesis and to suppress NF-κB activation and pro-inflammatory cytokines [25,26]. Moreover, rosmarinic acid in *Thunbergia laurifolia* has been reported as a free radical scavenger with antibacterial activity that also mitigates NF-κB and MAPK signaling [27,28]. In addition, vanillic acid in *Acanthus ebracteatus* has been reported to exhibit antibacterial, antioxidant, and anti-inflammatory activities via inhibition of oxidative stress and NF-κB activation [29]. In *Rhinacanthus nasutus*, plantamajoside has also been reported to possess antibacterial, antioxidant and anti-inflammatory activity via NF-κB and MAPK/PI3K-Akt modulation [30,31]. This difference in secondary metabolite profiles may be attributed to variations in plant material, extraction conditions, and analytical sensitivity, all of which can influence phytochemical detection. Although some active compounds were absent in this study, this does not necessarily indicate their complete absence in the sample, but rather suggests that their concentration may have been below the detection limit or affected by experimental conditions [32].

For antibacterial activity, the four selected species extracts demonstrated the ability to inhibit and kill skin pathogenic bacteria at varying concentrations—particularly the inhibition of methicillin-resistant *Staphylococcus aureus* (MRSA) observed in this study since MRSA is of considerable clinical importance due to its role in severe and drug-resistant skin infections. The difference in the antimicrobial properties among the extracts could be due to the secondary metabolite composition, as well as the mechanism of action of their bioactive constituents. The results in this research correspond to previous studies: Suriyaprom et al. [33] reported that three species in the family Acanthaceae including *Clinacanthus nutans*, *T. laurifolia*, and *A. ebracteatus* extracted with 95% ethanol inhibited some oral pathogenic bacteria. The chloroform extract of AP showed antimicrobial activity against Gram-positive and Gram-negative pathogenic bacteria [34]. Furthermore, the ethanolic extract of RN showed antibacterial potency in Gram-positive bacteria including *Bacillus cereus*, *B. atrophaeus* subsp. *globigii*, *B. subtilis*, *S. aureus*, *S. mutans*, *S. epidermidis*, and *C. acnes* [35,36]. Interestingly, although no clear zones of inhibition were observed in the agar well diffusion assay for some extracts, antibacterial activity was clearly demonstrated in both MIC and MBC assays. The absence of inhibition zones does not indicate a lack of antimicrobial activity but may instead reflect the physicochemical limitations of the diffusion method. The porous gel matrix of agar may restrict the diffusion of bioactive compounds, particularly those with high molecular weight or those that are non-polar or amphiphilic in nature. In contrast, in the broth microdilution method, the extracts directly interact with bacteria causing the facilitation of more efficient interaction between bioactive compounds and microbial targets.

Although plant extracts require higher doses than antibiotics to inhibit bacteria, the multi-targeting mechanism of phytochemicals from plant extracts might have the potential to reduce the development of bacterial resistance when compared to antibiotic usage [37,38]. Plant extracts might kill bacteria through several mechanisms including disruption of cell membranes, inhibition of essential enzymes, interference with nucleic acid synthesis, and modulation of quorum-sensing pathways. Specifically, medicinal plant polyphenols have been widely recognized for exerting these potent antimicrobial mechanisms [39]. Due to this multi-target mode of action, plant-derived compounds are less likely to induce bacterial resistance compared to antibiotics, which typically act on a single specific target. Therefore, plant extracts play an important role in combating antimicrobial resistance [40,41].

In anti-inflammatory activities, lipopolysaccharide (LPS) was selected to stimulate the inflammatory response in RAW 264.7 cells. This model organism is widely used to evaluate the anti-inflammatory potential of various compounds. LPS activates intracellular signaling pathways such as NF-κB and MAPKs which subsequently promote the production of inflammatory mediators. These transformations transform RAW 264.7 cells from a resting state into an activated macrophage state. In this study, treatment with Acanthaceae extracts resulted in the downregulation of the inflammatory gene under both pre-treatment and post-treatment conditions. In the pre-treatment model, cells were treated with plant extracts before LPS stimulation, allowing evaluation of their preventive effects. This research revealed that TL at 60 µg/mL (TL-60) showed the greatest suppression of *COX-2*, *TNF-α*, and *NF-κB* gene expression, while AP at 60 µg/mL (AP-60) demonstrated the most potent downregulation of *iNOS*. The greatest suppression of *IL-1β* was observed with RN at a concentration of 15 µg/mL (RN-15), whereas AE at 60 µg/mL (AE-60) exhibited the strongest downregulation of *IL-6*. These findings are consistent with previous studies. Li et al. [42] reported that andrographolide suppresses LPS-induced activation of the NF-κB/MAPK signaling pathway in macrophages, thereby inhibiting the production of pro-inflammatory cytokines such as TNF-α, IL-6, and IL-1β. Based on previous studies [43], the suppression of gene expression may be attributed to the secondary metabolites present in the extract, which could interfere with key regulatory steps in inflammatory signaling pathways, such as phosphorylation or nuclear translocation of NF-κB, ultimately leading to reduced expression of downstream target genes.

In the post-treatment model, LPS was exposed to macrophage cells to stimulate the inflammation, followed by treatment with plant extracts. The results showed that TL extract at a concentration of 60 ug/mL exhibited the greatest suppression of *iNOS* and *COX-2*. The most potent suppression of *IL-1β* and *NF-κB* was observed under treatment with RN extract at 15 ug/mL, while the AE extract exhibited the most potent downregulation of *IL-6* and *TNF-α.* Previous studies have reported that the major bioactive compounds in the family Acanthaceae have anti-inflammatory potency. In particular, phenolic compounds in TL, such as rosmarinic acid and chlorogenic acid, retain the capacity to suppress inflammatory mediators even after LPS-induced signaling has been initiated. This research showed that AE plant exhibited anti-inflammatory activity, which is in agreement with previous reports indicating that AE extract at 250 µg/mL inhibited the expression of pro-inflammatory cytokines in immune cells. The extract suppressed the release of TNF-α and nitric oxide as well as the downregulation of *IL-1β* [22]. The reduction in gene expression observed under this model may arise from the ability of secondary metabolites in the extracts to interfere with active NF-κB complexes that have already translocated to the nucleus, or to destabilize *iNOS* mRNA transcripts that have already been induced. However, the overall degree of suppression was generally lower than that observed under pre-treatment conditions, reflecting the greater mechanistic challenge of reversing an established inflammatory response.

Nitric oxide (NO) is an important mediator of physiological homeostatic processes and also functions as a cytotoxic defense molecule against infectious agents. However, overproduction of NO contributes to its role as a pro-inflammatory mediator that promotes inflammation [44,45]. In the present study, NO production was evaluated using both pre-treatment and post-treatment models. The results demonstrated that Acanthaceae extracts exhibited a greater inhibitory effect on NO production under pre-treatment conditions than under post-treatment conditions. This suggests that the secondary metabolites in these extracts may be more effective in preventing the initiation of iNOS-mediated NO synthesis than in suppressing an activated inflammatory response. All extracts significantly inhibited NO production when compared to the LPS-treated control group. These findings are consistent with previous reports demonstrating that extracts of AP, AE, TL and RN inhibit NO production in LPS-stimulated macrophages. Senghoi et al. [46] reported that TL leaf extract effectively suppresses NO production in LPS-induced RAW 264.7 macrophages. The reduction in NO levels observed in this study is consistent with the downregulation of *iNOS* gene expression, suggesting that the extracts may exert anti-inflammatory effects at the transcriptional level. Inhibition of *iNOS* expression during LPS stimulation likely contributes to decreased NO production in macrophages [47]. The reduction in NO production and the suppression of pro-inflammatory gene expression might be associated with oxidative stress regulation and intracellular signaling cascades. As demonstrated by the antioxidant assays in this study, the Acanthaceae extracts are rich in phenolic compounds with strong free radical scavenging abilities that may mitigate oxidative stress. Reactive oxygen species (ROS) are known activators of both the MAPK and NF-κB signaling cascades [48]. This antioxidant capacity could hypothetically attenuate the activation of these inflammatory pathways, thereby suppressing the transcription of downstream pro-inflammatory mediators [49].

Overall, these findings indicate that Acanthaceae plant extracts containing various secondary metabolites contribute to antioxidant, anti-skin pathogenic bacteria, and anti- inflammatory activities. In particular, this research indicates that extracts from plants in the family Acanthaceae suppress the mRNA expression of *iNOS*, *COX-2*, cytokines, and *NF-κB*, as well as inhibiting NO production. Therefore, the anti-inflammatory activity of Acanthaceae plant extracts is consistent with the possible regulation of NF-κB-associated inflammatory responses.

Despite the promising biological activities demonstrated in this study, there are still some limitations. The crude extracts contain various secondary metabolites, which limit the ability to determine active constituents of the exact that are responsible for the bioactivities when compared to purified compounds. Moreover, quality control of commercially obtained plant materials is required. In addition, in vitro models and a single murine macrophage cell line may not entirely represent human physiological responses.

Although the observed concentrations exhibit good bioactivity, their practical relevance for topical formulations requires further study on skin permeability and formulation compatibility. Therefore, future research utilizing in vivo models, different cell types, and topical formulation testing is necessary to confirm their suitability for skin treatments.

## 4. Materials and Methods

### 4.1. Reagents and Chemicals

Various analytical-grade reagents and chemicals were purchased from commercial suppliers. 2,2-diphenyl-1-picrylhydrazil (DPPH), 2,2′-azinobis-(3-ethylbenzothiazolin-6 sulfonic acid) (ABTS), 6-hydroxy-2,5,7,8-tetramethyl-chroman-2-carboxylic acid (Trolox), gallic acid monohydrate and vancomycin were purchased from Sigma-Aldrich (St. Louis, MO, USA). Folin–Ciocalteu reagent and TPTZ (2,4,6-tri(2-pyridyl)-s-triazine) were obtained from Merck (Billerica, MA, USA). Quercetin was obtained from Merck (Billerica, MA, USA). Aluminum chloride, ethanol, methanol, potassium acetate, and sodium carbonate were acquired from RCl Labscan (Bangkok, Thailand). Microbiological culture media, including Mueller Hinton broth (MHB) and Brain Heart Infusion (BHI) broth were purchased from HiMedia (Mumbai, Maharashtra, India). Dulbecco’s modified Eagle’s medium (DMEM) from Gibco (Grand Island, NY, USA) was used for RAW 264.7 macrophage cell culture. Gentamicin and MTT were purchased from Bio Basic (Toronto, ON, Canada). ReverTra Ace^®^ qPCR RT master mix was purchased from TOYOBO (Osaka, Japan). Molecular biology kits and reagents, including SensiFAST™ SYBR^®^ No-ROX Kit, was purchased from BIOLINE (London, UK). The TRIzol reagent was acquired from Invitrogen (Carlsbad, CA, USA).

### 4.2. Materials

The dry powder of four species of plant leaves from the family Acanthaceae including AP, TL, AE and RN were bought from Lanna Herbs, a store located in Warorot Market in Chiang Mai province, Thailand, in October 2024. The botanical identities of the materials were authenticated by Dr. Wittaya Pongamornkul, an ethnobotanist and botanist at the Research and Conservation Department, Queen Sirikit Botanic Garden Herbarium (QSBG Herbarium). The voucher specimens were deposited at the Queen Sirikit Botanic Garden Herbarium (QSBG), Chiang Mai, Thailand. The voucher specimen numbers are as follows: AP (WP9673), TL (WP9675), AE (WP9674), and RN (WP9672).

### 4.3. Extraction

The extraction method was performed following the previous extraction method [50]. Each sample was soaked in 95% ethanol in a ratio of 1:10 *w*/*v* at room temperature for 72 h. The solutions were filtered using No. 1 Whatman filter papers and then concentrated using a rotary evaporator (Heidolph, Schwabach, Germany) and freeze-dried on a lyophilizer (LABCONCO, Kansas City, MO, USA). The crude extracts were stored at −20 °C until they were used in experiments. The extract yield was calculated using the following equation.

Extraction yield (%) = Dry weight of extract obtained × 100/Weight taken of the dry sample

### 4.4. Determination of Total Phenolic and Flavonoid Contents

#### 4.4.1. Total Phenolic Contents

Determination of the total phenolic content in the plant extracts was performed in 96-well plates according to the Folin–Ciocalteu method adapted from Johnson et al. [51]. A 20 μL of sample was mixed with 100 μL of 10% (*v*/*v*) Folin–Ciocalteu solution and incubated in the dark for 10 min. After incubation, 100 μL of 7.5% (*w*/*v*) sodium carbonate was added, and the mixture was further incubated for 10 min under the dark condition. The absorbance was measured at 765 nm using a microplate reader (DYNEX Technologies, Chantilly, VA, USA). Total phenolic compound content was expressed as mg GAE/100 g extract by comparing to a gallic acid standard curve (3.125–400 μg/mL).

#### 4.4.2. Total Flavonoid Contents

Determination of the total flavonoid content in the plant extracts was evaluated using the colorimetric aluminum chloride assay. The method, performed in 96-well plates, was adapted from Sari et al. [52]. A total of 50 μL of extract was combined with 20 μL of 10% aluminum chloride; 100 μL of methanol, incubating for 3 min; and after that 20 μL of 1 M sodium acetate and 20 μL of methanol were added. Then, the mixture was measured for absorbance at 430 nm with a microplate reader after incubating in the dark for 40 min. Total flavonoid compound content was reported as milligrams of quercetin equivalent per 100 g of extract (mg QE/100 g extract) by comparing to a quercetin standard curve (12.5–400 μg/mL).

### 4.5. Liquid Chromatography–Mass Spectrometry (LC-MS) Characterization

Phytochemical constituents of the selected Acanthaceae extracts were characterized using liquid chromatography coupled with quadrupole time-of-flight mass spectrometry (LC-QTOF-MS; 6545XT AdvanceBio LC/Q-TOF, Agilent Technologies, Santa Clara, CA, USA) [53]. Separation was achieved on an InfinityLab Poroshell 120 EC-C18 column (2.1 × 100 mm, 2.7 µm, 120 Å; Agilent Technologies, Santa Clara, CA, USA) with the column temperature maintained at 50 °C. The mobile phase consisted of water containing 0.1% (*v*/*v*) formic acid (solvent A) and acetonitrile containing 0.1% (*v*/*v*) formic acid (solvent B). A gradient elution was applied over 20 min at a flow rate of 0.4 mL/min with an injection volume of 10 µL.

Mass spectra were acquired in both positive and negative electrospray ionization (ESI) modes under a high-resolution condition. The ion source parameters were as follows: drying gas at 325 °C and 13 L/min, sheath gas at 275 °C and 12 L/min, nebulizer pressure at 45 psi, and capillary voltage of +4000 V/−3000 V. Scanning was performed across an *m*/*z* range of 40–1700 (MS1) and 25–1000 (MS2), with collision energies of 20 eV in positive and 10 eV in negative modes.

Raw data were processed using MS-DIAL version 5.3 (RIKEN CSRS, Yokohama, Kanagawa, Japan). Sulfadimethoxine served as the internal standard for normalization. Metabolites were tentatively annotated based on retention time (0.2–18 min), mass accuracy (mass error ≤ ±20 ppm), and MS/MS spectral matching against the in-house ESI(+/−) MS/MS spectral library of authentic standards, the Fiehn/Vaniya natural products library, and BMDMS-NP, as provided in MS-DIAL. Spectral matching was evaluated using the MS-DIAL total score, a composite similarity metric incorporating mass accuracy, the isotopic ratio, and MS/MS spectral matching [54]. Metabolites with a total score ≥ 1.70 were selected for detailed profiling.

### 4.6. Antioxidant Activities of Four Selected Species of the Family Acanthaceae

#### 4.6.1. 2,2-Diphenyl-1-Picrylhydrazyl (DPPH) Radical Scavenging Assay

The radical scavenging activity was measured using a 2,2-diphenyl-1-picrylhydrazil (DPPH) assay adapted from Prieto [55]. A total of 0.2 mM DPPH and samples in various concentrations (0.0195–0.625mg/mL) were dissolved in methanol. The experiment was performed in 96-well plates; 100 µL of DPPH was mixed with 100 µL samples in each well then kept in the dark for 30 min at room temperature. The absorbance was measured at 517 nm using a microplate reader. The percentage inhibition was calculated and the result presented as IC_50_. Trolox was used as a positive control. The radical scavenging activity of Acanthaceae extracts was compared to a Trolox standard curve (1.56–200 μg/mL) and the results were presented as milligram of Trolox equivalent per gram of extract (mg TAE/g extract).

#### 4.6.2. ABTS Radical Cation Decolorization Assay

ABTS cation stock solution was prepared by mixing 7 mM ABTS with 2.45 mM Potassium persulfate in a ratio of 1:0.5, then kept in the dark for 16 h. ABTS stock solution was diluted with deionized water to 0.7 at an absorbance of 734 nm, then 10 µL ABTS was mixed with 100 µL of each sample incubate at room temperature for 6 min. The percentage inhibition of absorbance at 734 nm was calculated [56]. The radical scavenging activity of Acanthaceae extracts was compared to a standard Trolox calibration curve (1.56–200 µg/mL) and the results were presented as milligrams of Trolox equivalent per gram of extract (mg TAE/g extract).

### 4.7. Antibacterial Activities of Four Selected Species of the Family Acanthaceae

#### 4.7.1. Skin Pathogenic Bacterial Strains

The bacterial strains used in this study comprised the Gram-positive species *Staphylococcus aureus* ATCC 25923, *Micrococcus luteus*, and *Cutibacterium acnes* DMST 14916, together with the Gram-negative species *Pseudomonas aeruginosa* ATCC 27853, all of which were kindly supplied by the SCB2711 Microbiology Laboratory, Department of Biology, Faculty of Science, Chiang Mai University, Thailand. In addition, a clinical isolate of methicillin-resistant S. aureus (MRSA) was obtained from the Clinical Microbiology Unit, Department of Medical Technology, Faculty of Associated Medical Sciences, Chiang Mai University, Thailand.

Bacteria (*S. aureus*, *P. aeruginosa*, MRSA and *M. luteus*) were cultured in Mueller Hinton broth (MHB) at 37 °C for 24 h. *C. acnes* were cultured in Brain Heart Infusion (BHI) at 37 °C for 72 h without oxygen. The optical density at 600 nm (OD_600_) of the bacterial suspensions was adjusted to 0.1 to obtain a concentration of 1 × 10^8^ CFU/mL [57].

#### 4.7.2. Agar Well Diffusion Assay

An agar well diffusion assay was employed to screen the antimicrobial activity of ethanolic extracts of the plant extracts against skin pathogenic bacteria [58]. The bacterial suspension was adjusted to a turbidity corresponding to an approximate concentration of 1 × 10^8^ CFU/mL. The cultured bacteria were swabbed onto agar plates. Wells were then punched using sterile cork borers. Each well was filled with 100 µL of either plant extracts or the negative control. Gentamicin and vancomycin were used as the positive control. Gentamicin was used as a positive control for *P. aeruginosa*, *C. acnes*, *M. luteus*, and *S. aureus.* Vancomycin was used as a positive control for MRSA. After incubation under appropriated conditions for each bacterial strain, the diameter of the inhibition zone was recorded in millimeters.

#### 4.7.3. Minimum Inhibitory Concentration (MIC) and Minimum Bactericidal Concentration (MBC)

The MIC and MBC were conducted using the broth microdilution method in a 96-well plate following the method of Clinical and Laboratory Standards [59] to determine the lowest concentration that could inhibit and kill bacteria, respectively. Culture media, 50 μL, was added to each well. The initial concentration of the tested extract was 250 mg/mL, which was then two-fold serially diluted. Then, 50 μL of bacterial suspension (1 × 10^6^ CFU/mL) was added to each well, yielding a final inoculum concentration of 5 × 10^5^ CFU/mL per well. The plate was incubated under suitable conditions. The MIC in the wells with no growth of bacteria were determined.

After the MIC determination, aliquots from all the wells which showed no visible bacterial growth underwent streak plating on MH or BHI agar plates and were incubated under suitable conditions. The MBC was defined as the lowest concentration of the extract that resulted in the absence of bacterial growth on the agar plates. Therefore, a visible bacterial colony after bacterial treatment was not observed [60,61].

### 4.8. Anti-Inflammatory Activities of Acanthaceae Extracts

#### 4.8.1. Determination of RAW 264.7 Cell Toxicity

The murine macrophage cell line RAW 264.7 (ATCC, Manassas, VA, USA) was maintained in Dulbecco’s modified Eagle medium (DMEM, Gibco, Grand Island, NY, USA) containing 10% fetal bovine serum (FBS), supplemented with 100 U/mL penicillin and 100 µg/mL streptomycin (Gibco), at 37 °C in an incubator with a humidified atmosphere of 5% CO_2_.

The cytotoxicity of the plant extract on the RAW 264.7 macrophage cell line was determined using a 3-(4,5-Dimethylthiazol-2-yl)-2,5-Diphenyltetrazolium Bromide (MTT) assay with some modified from Jesus et al. [62]. The cells were seeded at 100,000 cells/well, incubated in an incubator for 24 h, then treated with plant extracts in concentrations of 15.625–250 µg/mL for 24 h, and cell viability was determined by incubation with 2 mg/mL of MTT for 3 h; after that, the solution was removed and 150 µL of DMSO was added to dissolve the crystals. The absorbance of the solution was measured at 540 and 630 nm using a microplate reader. In this study, cell toxicity was determined from the metabolic activity of cells using an MTT assay. The extracts at concentrations that did not significantly reduce cell viability relative to the untreated control group were considered non-toxic. Moreover, extracts at non-toxic concentrations with cell viability above 90% were subsequently selected for evaluation of anti-inflammatory activity.

#### 4.8.2. Nitric Oxide

The RAW 264.7 cells were seeded at a density of 100,000 cells per well in a 96-well plate and incubated at 37 °C in a 5% CO_2_ incubator for 24 h. The macrophage cells were treated with the extracts for 2 study models including pre-treatment and post-treatment with the plant extract [47].

Pre-treatment: Treatment with the plant extract before stimulation with LPS

The media were discarded and the cell was treated with 50 µL of a non-toxic concentration of Acanthaceae extract (15, 30 and 60 µg/mL for AP, TL and AE as well as 3.75, 7.5, and 15 µg/mL for RN) for 1 h, then 50 µL of LPS was added (final concentration of 10 ng/mL) to stimulate inflammation in the cell and incubated in a suitable condition for 24 h. The Griess reaction assay was used to quantify nitric oxide concentration in the cell culture medium after incubation [63]. Briefly, 100 µL of cell culture medium was combined with an equal volume of Griess, prepared from equal parts of 1% (*w*/*v*) sulfanilamide in 5% (*v*/*v*) phosphoric acid and 0.1% (*w*/*v*) N-(1-naphthyl) ethylenediamine dihydrochloride, and the mixture was incubated at room temperature for 15 min. The absorbance was measured at 550 nm by a microplate reader.

Post-treatment: Treatment with the plant extract after stimulation with LPS

The media were then removed and 50 µL of LPS was added (final concentration of 10 ng/mL) to the cell for 1 h for LPS stimulation, then incubated with 50 µL of a non-toxic concentration of Acanthaceae extract (15, 30 and 60 µg/mL for AP, TL and AE as well as 3.75, 7.5, and 15 µg/mL for RN) for 24 h. The concentration of nitric oxide in the culture supernatant was determined by the Griess reaction assay.

The NO inhibition (%) = (A_control_ − A_sample_/A_control_) × 100

A_control_ = The absorbance of the negative control

A_sample_ = The absorbance of the sample

#### 4.8.3. Quantitative Reverse Transcription–Polymerase Chain Reaction (RT-qPCR)

The RAW 264.7 cells were seeded at a density of 1,000,000 cells per well in 6-well plates incubated at 37 °C in a 5% CO_2_ incubator for 24 h. The macrophage cells were treated with the extracts including pre-treatment and post-treatment. Then the total RNA of the cells was prepared using Trizol^®^ reagent in accordance with the manufacturer’s protocol.

Total RNA was reverse transcribed into complementary DNA (cDNA) using ReverTra Ace^®^ qPCR RT Master Mix according to the manufacturer’s instructions. The reverse transcription reaction was performed at 37 °C for 15 min, followed by 50 °C for 5 min and 98 °C for 5 min. The resulting cDNA was then subjected to RT-qPCR analysis using the SensiFAST™ SYBR^®^ No-ROX Kit with gene-specific primers listed in Table 6. Beta-actin (β-actin) served as the internal reference gene for normalization. The qPCR program consisted of an initial denaturation at 95 °C for 2 min, followed by 40 amplification cycles of denaturation at 95 °C for 5 s and combined annealing/extension at 65 °C for 30 s. The mRNA expression levels of the pro-inflammatory genes *COX-2*, *iNOS*, *IL-1β*, *IL-6*, *TNF-α*, and *NF-κB* were subsequently quantified by RT-qPCR [64].

### 4.9. Statistical Analysis

The results were indicated as mean ± standard deviation (S.D.) from three different experiments. The program Statistical Package for the Social Sciences version 29.0 for Windows (IBM SPSS Statistics) was used to analyze the results by one-way ANOVA with Duncan’s post hoc test and a statistical significance was set at *p* < 0.05.

## 5. Conclusions

This study confirms the benefit of the traditional use of four selected medicinal plants from the Acanthaceae family, including *Andrographis paniculata*, *Thunbergia laurifolia*, *Acanthus ebracteatus*, and *Rhinacanthus nasutus*, by demonstrating their biological efficacies. Through comprehensive LC-MS profiling, the diverse secondary metabolites in the extracts were linked to their potent antioxidant, antibacterial, and anti-inflammatory activities. Furthermore, all extracts exhibited significant antibacterial efficacy against skin pathogenic bacteria, highlighting their potential as promising alternative agents to antibiotics. Notably, the systematic evaluation of both pre-treatment and post-treatment models provided a comparative approach. The extracts effectively inhibited nitric oxide production and suppressed the mRNA expression of key inflammatory mediators, clearly revealing that they are significantly effective in preventing inflammatory initiation more than an established inflammatory response in LPS-stimulated RAW 264.7 macrophages.

These findings support the traditional use of Acanthaceae plants in Thai traditional medicine and highlight their potential as natural sources of antioxidant, antibacterial, and anti-inflammatory effects. Future studies should be focused on protein level expression and signaling pathways. Moreover, techniques such as Western blot analysis and ELISA for cytokine profiling should be performed to further elucidate the mechanisms of action. Furthermore, the therapeutic potential of these plant extracts should be evaluated for the management of skin infections and inflammatory conditions. Subsequent investigations should be concerned with biofilm inhibition and should incorporate the study of in vivo skin infection models.

## Figures and Tables

**Table 1:** Extraction yields of four selected species of the family Acanthaceae.

Plant Extracts	Weight of Extract (g)	Yield (% *w*/*w*)
AP	13.40	6.68
TL	12.38	6.18
AE	8.55	4.26
RN	4.25	2.12

**Table 2:** Total phenolic and flavonoid contents of four selected species of the family Acanthaceae.

Plant Extracts	Total Phenolic Content (mg GAE/100 g Extract)	Total Flavonoid Content (mg QE/100 g Extract)
AP	18.33 ± 1.08 ^d^	12.89 ± 1.04 ^d^
TL	153.97 ± 1.55 ^a^	60.17 ± 0.87 ^a^
AE	73.56 ± 1.25 ^b^	13.76 ± 0.39 ^c^
RN	32.07 ± 2.36 ^c^	27.91 ± 0.73 ^b^

Each value in the table is represented as mean ± SD (*n* = 3); ^a–d^ within the same column indicate significant differences (*p* < 0.05).

**Table 3:** Antioxidant activity of four selected species of the family Acanthaceae.

Plant Extracts	DPPH		ABTS	
	IC_50_ (mg/mL)	Antioxidant Activity (mg TAE/g Extract)	IC_50_ (mg/mL)	Antioxidant Activity (mg TAE/g Extract)
AP	0.67 ± 0.08 ^a^	20.43 ± 2.26 ^c^	4.22 ± 0.35 ^a^	2.82 ± 0.24 ^c^
TL	0.16 ± 0.02 ^d^	85.09 ± 11.70 ^a^	0.76 ± 0.07 ^c^	15.71 ± 1.34 ^a^
AE	0.33 ± 0.09 ^c^	43.80 ± 13.44 ^b^	1.43 ± 0.12 ^b^	8.34 ± 0.66 ^b^
RN	0.55 ± 0.04 ^b^	24.73 ± 1.74 ^c^	1.45 ± 0.01 ^b^	8.16 ± 0.05 ^b^

Each value in the table is represented as mean ± SD (*n* = 3); ^a–d^ within the same column indicate significant differences (*p* < 0.05).

**Table 4:** Diameter of inhibition zones of plant extracts from the Acanthaceae family at a concentration of 500 mg/mL.

Plant Species	Bacterial Strain Zone of Inhibition (mm)				
	*P. aeruginosa*	*C. acnes*	*M. luteus*	*S. aureus*	MRSA
AP	ND	ND	23.5 ± 0.6	15.0 ± 0.4	13.0 ± 0.4
TL	12.0 ± 0.8	ND	22.3 ± 1.1	17.9 ± 0.3	19.7 ± 0.5
AE	11.7 ± 0.8	ND	16.4 ± 0.9	14.7 ± 0.6	15.8 ± 0.3
RN	10.8 ± 0.7	ND	15.0 ± 0.4	16.3 ± 0.8	14.3 ± 0.3
Antibiotic (1 mg/mL)	31.2 ± 1.3	21.9 ± 0.4	41.7 ± 0.3	30.2 ± 0.3	23.3 ± 0.6

Each value in the table is represented as mean ± SD (*n* = 3); ND = zone of inhibition not detected. Gentamicin was used as a positive control for *P. aeruginosa*, *C. acnes*, *M. luteus*, and *S. aureus*. Vancomycin was used as a positive control for MRSA.

**Table 5:** Minimum inhibitory concentration (MIC) and minimum bactericidal concentration (MBC) of plant extracts from four selected species of the family Acanthaceae.

Plant Extracts	Concentration of Plant Extract (mg/mL)									
	*P. aeruginosa*		*C. acnes*		*M. luteus*		*S. aureus*		MRSA	
	MIC	MBC	MIC	MBC	MIC	MBC	MIC	MBC	MIC	MBC
AP	125.00	125.00	0.98	0.98	62.50	62.50	62.50	62.50	62.50	62.50
TL	31.25	31.25	1.95	1.95	31.25	31.25	3.91	3.91	7.81	7.81
AE	15.63	15.63	3.91	3.91	31.25	31.25	15.63	15.63	7.81	7.81
RN	62.50	62.50	3.91	3.91	31.25	31.25	7.81	7.81	15.63	15.63
Antibiotic (µg/mL)	0.98	0.98	1.95	1.95	1.95	1.95	0.49	0.49	0.98	0.98

MIC or MBC values represent results from three independent replicates (*n* = 3). Gentamicin was used as a positive control for *P. aeruginosa*, *C. acnes*, *M. luteus*, and *S. aureus*. Vancomycin was used as a positive control for MRSA.

**Table 6:** Primer sequences of inflammatory genes used for RT-qPCR.

Gene		Sequences	References
*iNOS*	Forward	5′ TTCCAGAATCCCTGGACAAGC 3′	[65]
	Reverse	5′ TGGTCAAACTCTTGGGGTTCG 3′	
*COX-2*	Forward	5′ AGAAGGAAATGGCTGCAGAA 3′	[65]
	Reverse	5′ GCTCGGCTTCCAGTATTGAG 3′	
*TNF-α*	Forward	5′ AGCCCCCAGTCTGTATCCTTC 3′	[65]
	Reverse	5′ CATTCGAGGCTCCAGTGAATTCG 3′	
*IL-6*	Forward	5′ GCTGGAGTCACAGAAGGAGTG 3′	[66]
	Reverse	5′ GCATAACGCACTAGGTTTGCC 3′	
*IL-1β*	Forward	5′ CATTGTGGCTGTGGAGAAGCT 3′	This study
	Reverse	5′ CATGAGTCACAGAGGATGGGC 3′	
*NF-κB* (p105/p50)	Forward	5′ GAAATTCCTGATCCAGACAAAAACTGG 3′	[67]
	Reverse	5′ ATCACTTCAATGGCCTCTGTGTAG 3′	
*β-actin*	Forward	5′ TGCTGTCCCTGTATGCCTCTG 3′	[68]
	Reverse	5′ GCTGTAGCCACGCTCGGTCA 3′	

Primers were designed in this study using NCBI Primer-BLAST [69] based on GenBank accession no. NM_008361.4.

## Data Availability

The data presented in this study are available on request from the corresponding author due to privacy.

## Supplementary Materials

The following supporting information can be downloaded at: https://www.mdpi.com/article/10.3390/plants15172650/s1, Table S1: Tentatively annotated metabolites in negative ionization modes detected in four selected species extracts from the Acanthaceae family by LC-MS, showing retention time, mass-to-charge ratio, mass error, adduct type and total score; Table S2: Tentatively annotated metabolites in positive ionization modes detected in four selected species extracts from the Acanthaceae family by LC–MS, showing retention time, mass-to-charge ratio, mass error, adduct type and total score.

## Abbreviations

The following abbreviations are used in this manuscript:

AP	*Andrographis paniculata*
TL	*Thunbergia laurifolia*
AE	*Acanthus ebracteatus*
RN	*Rhinacanthus nasutus*
