# Supplementary material for: Inhibitory Efficacy of Medicinal Plant Extracts from the Family Acanthaceae Against Skin Pathogenic Bacteria, Antioxidant and Anti-Inflammatory Activities
Source: Plants (Basel). 2026 Aug 29;15(17):2650. doi: 10.3390/plants15172650 (PMC13567256; doi:10.3390/plants15172650)
Supplement: Supplementary file 1 [file plants-15-02650-s001.zip › plants-4454383-supplementary.pdf]

Supplementary Materials

Table S1. Tentatively annotated metabolites in negative ionization modes detected in 4 selected species extracts from Acanthaceae family by LC–MS, showing retention time, mass-to-charge ratio, mass error, adduct type and total score.

Metabolite	RT (min)	m/z	Mass error (ppm)	Adduct type	Total score
Catechol	2.921	109.02944	-4.22	$[M-H]^-$	1.777
Vanillic acid	3.028	167.03490	-4.25	$[M-H]^-$	1.774
Caffeic acid	4.082	179.03526	-1.45	$[M-H]^-$	1.765
12-Hydroxyoctadecanoic acid	14.881	299.26016	-3.24	$[M-H]^-$	1.764
Catechol	0.709	109.02933	-9.45	$[M-H]^-$	1.756
6,7-Dihydroxycoumarin	4.075	177.01947	-3.73	$[M-H]^-$	1.749
Caffeic acid	5.937	179.03513	-3.80	$[M-H]^-$	1.742
Caffeic acid	6.039	179.03503	-3.24	$[M-H]^-$	1.741
D-(-)-Quinic acid	0.689	191.05688	-6.75	$[M-H]^-$	1.738
Naringenin	7.668	271.06250	-4.83	$[M-H]^-$	1.736
Dihydrocoumarin	5.524	147.04491	-4.83	$[M-H]^-$	1.734
Kaempferol	6.951	285.04050	1.37	$[M-H]^-$	1.733
Azelaic acid	6.057	187.09764	-0.21	$[M-H]^-$	1.732
2-hydroxyquinoline	5.638	144.04512	2.57	$[M-H]^-$	1.731
Umbelliferone	7.614	161.02519	-3.04	$[M-H]^-$	1.725
FA 18:1+1O	13.250	297.24338	2.05	$[M-H]^-$	1.725
3-(2-Hydroxyphenyl) propanoate	5.537	165.05588	-7.51	$[M-H]^-$	1.724
Pygenic acid A b	15.140	471.34738	0.25	$[M-H]^-$	1.723
Emodin	7.684	269.04590	-1.38	$[M-H]^-$	1.722
Chlorogenic Acid	3.886	353.08960	-5.10	$[M-H]^-$	1.720
2-hydroxyquinoline	3.654	144.04562	-0.90	$[M-H]^-$	1.719
Apigenin-7-O-glucuronide	5.917	445.07776	-0.34	$[M-H]^-$	1.718
3-Hydroxypyridine	0.709	94.02996	-6.91	$[M-H]^-$	1.717
y-Linolenic acid	12.730	277.21695	3.75	$[M-H]^-$	1.714

6-Geranylnaringenin	13.217	407.18655	4.05	[M-H]⁻	1.714
2-Hydroxybenzaldehyde	4.260	121.02943	5.54	[M-H]⁻	1.708
Protocatechuic aldehyde	3.546	137.02501	-9.56	[M-H]⁻	1.706
9-(2,3-dihydroxypropoxy)-9-oxononanoic acid	5.819	261.13446	1.30	[M-H]⁻	1.705
4-hydroxy-benzoate	5.948	137.02443	-3.87	[M-H]⁻	1.705
Galangin	11.129	269.04544	4.31	[M-H]⁻	1.702
Rosmarinic acid	5.355	359.07797	-2.03	[M-H]⁻	1.700

Table S2. Tentatively annotated metabolites in positive ionization modes detected in 4 selected species extracts from Acanthaceae family by LC–MS, showing retention time, mass-to-charge ratio, mass error, adduct type and total score.

Metabolite	RT (min)	m/z	Mass error (ppm)	Adduct type	Total score
2'-O-methylisoliquiritigenin	7.858	293.08206	-7.06	[M+Na]⁺	1.876
1,3,8-trihydroxy-6-methylanthra-9,10-quinone	7.689	271.06073	-2.69	[M+H]⁺	1.864
5,7-dihydroxy-3-phenyl-4H-chromen-4-one	9.639	255.06691	12.15	[M+H]⁺	1.844
2-(5-methoxy-4,8,8-trimethyl-2-oxo-2,8,9,10-tetrahydropyrano[2,3-f]chromen-3-yl)-N-(pyridin-2-yl)acetamide	4.569	409.18445	-10.90	[M+H]⁺	1.827
1,3,8-trihydroxy-6-methylanthra-9,10-quinone	5.901	271.06116	-4.28	[M+H]⁺	1.826
Fuberidazole	3.398	185.07129	-1.57	[M+H]⁺	1.814
Coumarin	5.809	147.04509	-6.73	[M+H]⁺	1.810
Norharman	4.495	169.07661	-3.61	[M+H]⁺	1.810
Kynurenic Acid	3.668	190.04976	0.74	[M+H]⁺	1.805
3-Hydroxypyridine	0.678	96.04443	5.00	[M+H]⁺	1.804
Umbelliferone	5.091	163.03949	-3.01	[M+H]⁺	1.795
Aurantiamide acetate	11.007	445.21738	-12.96	[M+H]⁺	1.791
Adenine	2.406	136.06207	-5.73	[M+H]⁺	1.786
Quercetin	4.624	303.05072	-2.41	[M+H]⁺	1.785
Sucrose	0.742	365.10638	-3.75	[M+Na]⁺	1.783

2-Naphthylamine	3.996	144.08177	-6.73	$[M+H]^+$	1.782
Quercetin	5.145	303.05090	-3.00	$[M+H]^+$	1.780
Denatonium	5.480	325.22818	-2.24	$[M]^+$	1.776
Limonin	9.212	471.20200	-4.22	$[M+H]^+$	1.772
Tectorigenin	6.230	301.07172	-5.68	$[M+H]^+$	1.770
Kuwanon E	8.762	447.17847	3.40	$[M+Na]^+$	1.767
1,8-dihydroxy-3-methylanthra-9,10-quinone	11.374	255.06541	-1.61	$[M+H]^+$	1.767
Harpagide	2.702	387.13126	-10.98	$[M+Na]^+$	1.756
LysoPC(0:0/18:0)	13.880	524.37408	-0.80	$[M+H]^+$	1.755
(Z)-5,8,11-trihydroxyoctadec-9-enoic acid	9.247	353.23090	-4.84	$[M+Na]^+$	1.754
7-O-Methylchrysin	7.785	269.08176	-6.58	$[M+H]^+$	1.753
1,3,8-trihydroxy-6-methylanthra-9,10-quinone	7.315	271.06033	-1.22	$[M+H]^+$	1.752
Praeruptorin E	11.405	451.17178	-3.92	$[M+Na]^+$	1.749
7-hydroxy-2-(4-hydroxyphenyl)-5-methoxy-2,3-dihydro-4h-chromen-4-one	5.384	287.09192	-1.81	$[M+H]^+$	1.747
Gentisyl alcohol	3.639	141.05466	-4.04	$[M+H]^+$	1.742
7-hydroxy-2-(4-hydroxyphenyl)-5-methoxy-2,3-dihydro-4h-chromen-4-one	4.375	287.09106	1.18	$[M+H]^+$	1.742
2-(3,4-dihydroxyphenyl)-5,7-dihydroxy-3-(((2S,3R,4S,5S,6R)-3,4,5-trihydroxy-6-((((2R,3R,4R,5R,6S)-3,4,5-trihydroxy-6-methyltetrahydro-2H-pyran-2-yl)oxy)methyl)tetrahydro-2H-pyran-2-yl)oxy)-4H-chromen- 4-one trihydrate	5.164	633.14478	-7.53	$[M+Na]^+$	1.740
2-hydroxy-3-{[3,4,5-trihydroxy-6-(hydroxymethyl)oxan-2-yl]oxy}propyl (9Z,12Z,15Z)-octadeca-9,12,15-trienoate	13.613	515.32166	-3.20	$[M+H]^+$	1.739
Luteolin-7-glucoside	6.323	449.10757	0.76	$[M+H]^+$	1.737
Plantamajoside	4.709	663.19086	-1.30	$[M+Na]^+$	1.733
14-deoxy-11,12-dihydroandrographolide	9.069	355.19351	-9.88	$[M+Na]^+$	1.732
7-hydroxy-2-(4-hydroxyphenyl)-5-methoxy-2,3-dihydro-4h-chromen-4-one	8.631	287.09418	-9.68	$[M+H]^+$	1.732
Dihydroberberine	5.438	338.13846	4.58	$[M+H]^+$	1.732
Denatonium	6.819	325.22800	-1.69	$[M]^+$	1.730

2-(2-methoxy-phenyl)-5-oxo-tetrahydro-furan-3-carboyl-lupinine	6.698	388.21155	-4.02	$[M+H]^+$	1.728
Luteolin	5.559	287.05600	-3.41	$[M+H]^+$	1.727
Icariside F2	4.223	403.15817	4.54	$[M+H]^+$	1.727
Bullatine G	10.356	358.22842	4.44	$[M+H]^+$	1.723
Andrographolide	6.450	373.20056	-1.47	$[M+Na]^+$	1.722
Trigonelline	0.687	138.05525	1.67	$[M+H]^+$	1.722
Schisanhenol	9.635	425.19122	-2.87	$[M+Na]^+$	1.721
LysoPC (0:0/18:0)	13.874	546.35223	2.69	$[M+Na]^+$	1.721
Neochlorogenic acid	3.866	355.10263	-0.76	$[M+H]^+$	1.718
5,7,4'-Trihydroxy-8-methylflavanone	9.688	287.09189	-6.58	$[M+H]^+$	1.717
Fludrocortisone	13.401	423.21628	3.38	$[M+H]^+$	1.716
Kuwanon E	4.746	447.18430	-9.64	$[M+Na]^+$	1.713
NCGC00385412-01_C21H32O10_2,4-Pentadienoic acid, 5-[(1R,3S,5R,8S)-3-(beta-D-glucopyranosyloxy)-8-hydroxy-1,5-dimethyl-6-oxabicyclo[3.2.1]oct-8-yl]-3-methyl-, (2Z,4E)-	3.798	467.18805	2.03	$[M+Na]^+$	1.713
Nicotinic acid	0.751	124.03925	-6.05	$[M+H]^+$	1.712
Denatonium	0.703	325.22766	-0.65	$[M]^+$	1.712
Tectorigenin	6.543	301.07056	-1.83	$[M+H]^+$	1.711
Asperphenamate_130119	12.370	507.23047	-4.87	$[M+H]^+$	1.710
Gomisin H	7.202	419.20529	11.21	$[M+H]^+$	1.710
14-deoxy-11,12-dihydroandrographolide	8.739	355.19052	-1.46	$[M+Na]^+$	1.709
andrographolide	7.029	373.20261	-6.97	$[M+Na]^+$	1.709
Lycorine	5.086	310.10852	4.74	$[M+Na]^+$	1.708
Sophoraflavonoloside	4.628	633.14276	-4.34	$[M+Na]^+$	1.708
Bruceine A	5.150	545.20013	-0.22	$[M+Na]^+$	1.704
Pinocembrin	10.916	257.08099	-0.35	$[M+H]^+$	1.703
Alpinetin Methyl Ether	10.088	285.11575	-14.87	$[M+H]^+$	1.702
17-Hydroxyisolathyrol	6.770	373.19937	1.71	$[M+Na]^+$	1.702
4,5-Di-O-caffeoylquinic acid	5.930	539.11639	6.70	$[M+Na]^+$	1.701
